# Rethinking Nutrition in Chronic Kidney Disease: Plant Foods, Bioactive Compounds, and the Shift Beyond Traditional Limitations: A Narrative Review

**DOI:** 10.3390/foods14193355

**Published:** 2025-09-27

**Authors:** Nerea Nogueira-Rio, Alicia del Carmen Mondragon Portocarrero, Alexandre Lamas Freire, Carlos Manuel Franco, Ahmet Alperen Canbolat, Sercan Karav, Jose Manuel Miranda Lopez

**Affiliations:** 1 Department of Analytical Chemistry, Nutrition and Food Science, School of Veterinary Sciences, University of Santiago de Compostela, 27002 Lugo, Spain; nerea.nogueira@rai.usc.es (N.N.-R.); alicia.mondragon@usc.es (A.d.C.M.P.); alexandre.lamas@usc.es (A.L.F.); carlos.franco@usc.es (C.M.F.); 2 Department of Molecular Biology and Genetics, Çanakkale Onsekiz Mart University, Çanakkale 17000, Türkiye; ahmetalperencanbolat@stu.comu.edu.tr (A.A.C.); sercankarav@comu.edu.tr (S.K.)

**Keywords:** dietary fiber, resistant starch, bioactive compounds, hyperphosphatemia, flavonoids, fusetin, photerin, sulforaphane

## Abstract

The incidence of chronic kidney disease (CKD) has increased worldwide in recent years. Many factors can contribute to the progression of CKD, some of which are dietary patterns. Adequate control of protein, phosphorus, potassium, and sodium intake can significantly slow the progression of CKD. Most studies and nutritional guidelines addressing the care of people with CKD have focused primarily on dietary recommendations regarding macronutrient intake and the restriction of individual micronutrients. Traditionally, the consumption of fiber-rich fruits and vegetables has been restricted in patients with CKD to combat hyperkalemia. Among the reasons often given for this restriction are concerns about their high potassium and phosphorus contents. Limiting the intake of whole grains in CKD patients has also been recommended. However, findings indicate that phosphorus in plant foods is not fully absorbed in humans. Potassium contribution from vegetables can be reduced by culinary treatments, and when highly insoluble fiber is present in vegetables, it promotes potassium excretion through the intestine, which could help control the risk of hyperkalemia in CKD patients. Other recent findings have shown beneficial effects of vegetable bioactive compounds and resistant starch on CKD patients. The aim of the present review was to compile and discuss traditional recommendations for the use of plant-based foods for patients with CKD, as well as the mechanisms through which such foods may contribute to improving CKD progression.

## 1. Introduction

Chronic kidney disease (CKD) is a prevalent and complex disease that is rapidly becoming a growing public health problem. The worldwide prevalence of CKD is increasing; it currently affects 10–15% of the population and is associated with high morbidity and mortality [1,2]. This recent increase has been attributed, in part, to the aging process that can be observed in the world population. The prevalence of CKD is 33% in adults older than 65 years [3]. There are several reasons for this high prevalence. On the one hand, aging is accompanied by reductions in muscle mass and function due to an increase in catabolic responses, a decrease in anabolic responses in muscle metabolism, a decrease in physical activity, and a reduction in energy balance intake [3]. In addition to aging, other concomitant risk factors, such as hypertension, diabetes, obesity, hyperlipidemia, smoking, and inadequate dietary patterns, can also be partly attributed to the increase in CKD rates [4,5].

The impact of CKD on human health extends far beyond simple renal complications, with a range of consequences for other organs and systems, such as cardiovascular disease (CVD), anemia, bone disorders, and metabolic disturbances, including insulin resistance, dyslipidemia, chronic inflammation, and metabolic acidosis, all of which contribute to increased morbidity and mortality in CKD patients [6,7]. In addition, CKD is characterized by the accumulation of uremic toxins (uremia), anomalies in amino acid and homocysteine metabolism, vitamin D deficiency, skeletal muscle dysfunction with reduced exercise tolerance, and loss of lean body mass leading to cachexia [7]. Other contributing factors include erythropoietin resistance, oxidative stress, and vascular calcification [2,6,7]. Paradoxically, consequences of overnutrition, such as obesity and hyperlipidemia, have been shown to improve the survival of CKD patients [8,9]. Inflammation influences not only renal function, but also the development of the systemic comorbidities described above, including CVD, diabetes mellitus, hypertension, and anemia. Moreover, chronic low-grade systemic inflammation, promoted by social, environmental, and lifestyle factors, has been associated with additional conditions such as cancer, non-alcoholic fatty liver disease, autoimmune disorders, and neurodegenerative diseases [2,10].

Dietary patterns play a pivotal role in modulating the inflammatory status of individuals, with evidence suggesting that dietary components can exert pro-inflammatory or anti-inflammatory effects [11]. To prevent the progression of CKD from a nutritional approach, protein restriction, adequate caloric intake, and correction of electrolyte abnormalities have traditionally been recommended [12], with a special focus on potassium, sodium, and phosphorus intake [3].

High protein intake contributes to glomerular hyperfiltration and elevated intraglomerular pressure, with consequent renal damage. In addition, a normoprotein or hyperprotein diet may potentiate uremic symptoms and hyperphosphatemia [8]. However, not all types of protein are equally harmful to these patients, but high dietary intake of vegetable protein, vitamin C, Mg, K, and n-6 polyunsaturated fatty acids is recommended [4]. Recommended protein intake is not the same for all CKD patients, depending on their glomerular filtration rate. Based on the KDIGO 2024 Clinical Practice Guidelines for Nutrition in CKD patients [13], a very-low-protein diet (0.3–0.4 g/kg body weight/d) supplemented with essential amino acids or ketoacid analogs is recommended for adults with CKD at risk of kidney failure, under close supervision,. For patients with CKD G3–G5 stage (nondialysis), a protein intake of 0.8 g/kg body is recommended, emphasizing the need to avoid a dietary protein intake of >1.3 g/kg body weight/day. However, for adult CKD patients on peritoneal dialysis and hemodialysis, a daily protein intake of 1.0–1.2 g/kg body weight/day is recommended [13].

The recently updated KDIGO 2024 Clinical Practice Guidelines for Nutrition in CKD patients also stated recommendations on adequate intake of dietary fiber from vegetal sources. Because of their cellulose content, plant foods are the main source of dietary fiber in Western countries [11], which, in the early stages of CKD, is associated with better overall health and renal outcomes [13]. In addition, adequate fiber intake decreases other concomitant risk factors, resulting in decreased body weight and improved blood pressure [14].

Among the usual foods in Western dietary patterns, there is generally a deficit in the intake of fiber sources such as fruits and vegetables [15]. In contrast, there is usually a high consumption of cereals and cereal-derived foods, such as bread. Bread is often considered a counterproductive food for patients with CKD, since it usually has a high sodium content and phosphorus additives are commonly added during its formulation [16]. The number of articles that have investigated the effects of vegetable foods, whole grains, and foods derived from cereals in patients with CKD is relatively low. Additionally, there has not been much research on the effects that some bioactive compounds from vegetable foods can exert in CKD patients through inflammatory regulation. The effects of other carbohydrate components from foods, such as resistant starch, may also have specific effects on CKD patients via modulation of the gut microbiota (GM).

**Figure 1:**
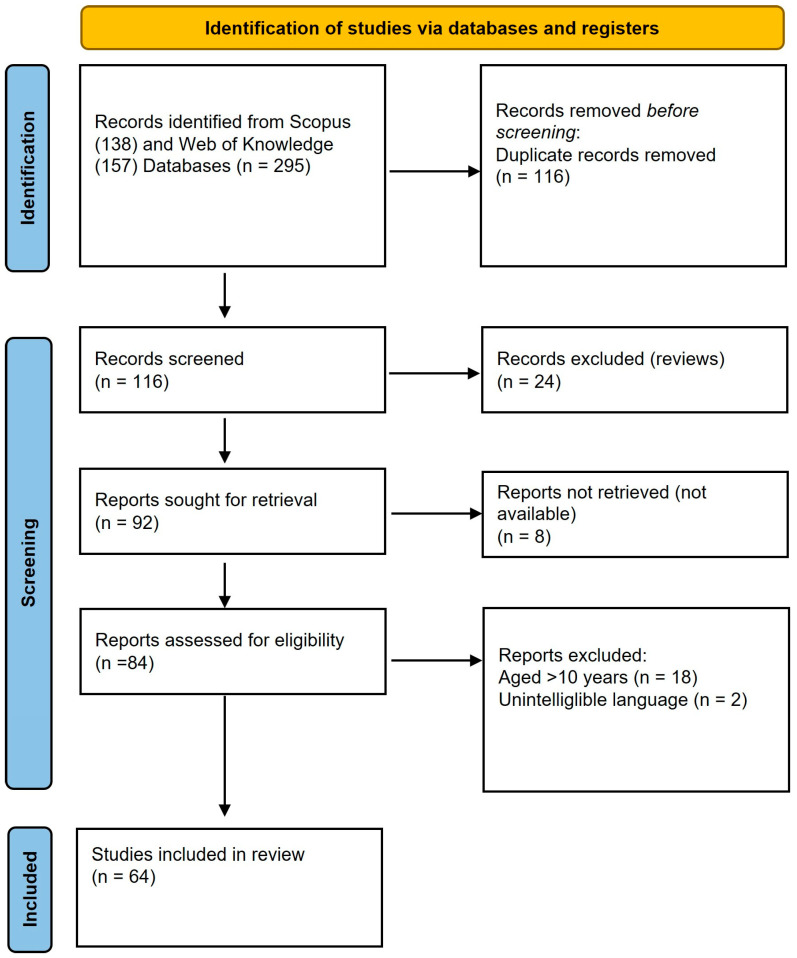
PRISMA 2020 flow chart of reviewed studies.

Therefore, this narrative literature search was conducted between April and June 2025 for all the available literature in the Web of Science and Scopus. A combination of the following search terms was applied: “Chronic kidney disease” in “title” and “dietary fiber”; “bioactive compounds”; “whole bread”; “whole grains”; and “resistant starch” in “topic” or “article title, abstract, keywords”. Finally, a total of 64 articles were selected and included in the review, as were 32 others that were included to contextualize the articles (Figure 1). The aim of this review was to compile and discuss traditional recommendations for the use of plant-based foods in patients with CKD, as well as to explore the mechanism through which these foods may contribute to slowing CKD progression.

## 2. Dietary Considerations for CKD Patients

Many factors can contribute to the progression of CKD, including excessive protein consumption, metabolic acidosis, weight gain, hypertension, and dyslipidemia, each of which is influenced by diet [17]. Although the crucial role of nutritional interventions in preventing or delaying the progression of CKD and its complications is well established [18], most studies and nutritional guidelines addressing the care of people with CKD focus mainly on dietary recommendations regarding macronutrient intake and the restriction of individual micronutrients [19]. In this context, adequate control of protein, phosphorus, potassium, and sodium intake can significantly slow the progression of CKD [20].

**Figure 2:**
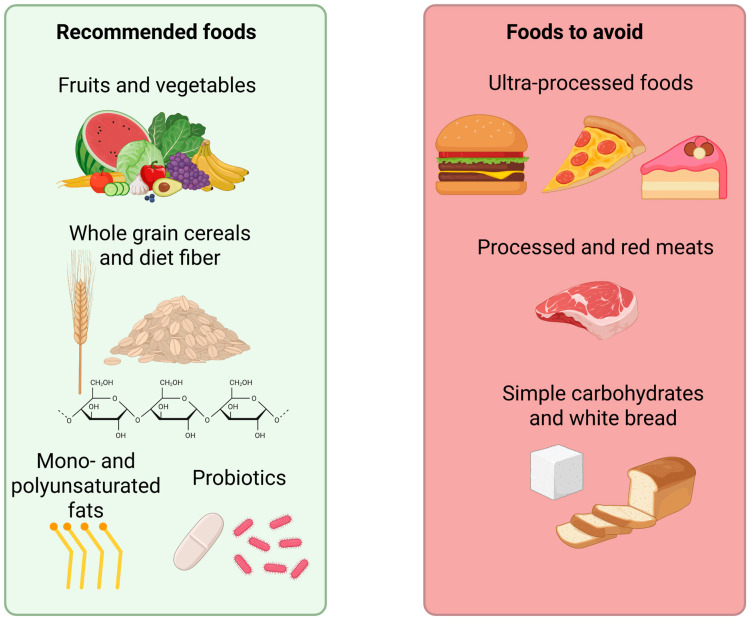
Recommended foods and foods to avoid chronic kidney disease.

The regular consumption of plant-based foods such as cereals, raw vegetables, cooked vegetables, and fresh fruit is among the main nutritional factors associated with the prevention of CKD [21]. Conversely, high consumption of processed meat, white bread, and other less healthy foods is associated with an increased risk of CKD episodes [21] (Figure 2). The consumption of ultra-processed foods (UPFs) is also associated with an increased risk of CKD [22]. The recommended energy intake is approximately 25–35 kcal/kg/day for all stages of CKD [16]. This energy should come mainly from healthy macronutrients, so simple carbohydrates (sugars) should be avoided, and complex carbohydrates rich in fiber should be consumed. For lipids, the consumption of monounsaturated and polyunsaturated fatty acids is recommended instead of trans fats or saturated fatty acids, which increase the risk of CVD [23].

Among dietary patterns, preliminary results from prospective studies have shown that better adherence to a Mediterranean or dietary approach to stop hypertension (DASH)-type diet is associated with a lower incidence of severe kidney damage and a lower risk of kidney function decline [24]. Recently, it was suggested that adherence to the DASH diet was inversely associated with the risk of end-stage renal disease in adults with CKD, particularly in people with diabetes [25].

Modulation of the gut microbiota (GM) is another area of growing interest in CKD dietary management. A Korean population-based study revealed that while the use of amino acids, protein, ginseng, and herbal supplements was associated with increased CKD incidence, probiotic consumption was correlated with significantly decreased CKD incidence [26]. This may be due to the potential of probiotics to improve the GM balance and gut epithelial maintenance, which are frequently disrupted in CKD patients due to low fiber intake, acidosis, and uremia [26].

Plant-based foods contain citrate, which helps buffer the metabolic acidosis typical of CKD and maintain acid–base balance. The KDIGO nutritional guidelines recommend high consumption of fruits and vegetables to counteract acid production and preserve residual kidney function [17]. Metabolic acidosis in CKD increases the production of angiotensin, aldosterone, and endothelin-1, all of which contribute to disease progression. Patients with a relatively high dietary acid load are at increased risk for osteopenia, poor growth, and renal failure [17]. In addition to high intake of plant-based foods, the typical acidosis of CKD can also be corrected by oral alkaline therapy involving the use of sodium-based alkalizing agents (Na+, Na+ citrate, and NaHCO_3_). However, these agents may worsen volume retention and hypertension in patients with CKD [8].

## 3. The Role of Phosphorus in the Diet of Chronic Kidney Disease Patients

The parathyroid glands play an essential role in CKD patients by regulating serum calcium and phosphorus levels through the secretion of parathyroid hormone (PTH). Phosphate in the human body is mainly stored in bones (85%) and soft tissues (14%), with only 1% circulating in the blood, where normal levels range from 0.81 to 1.45 mmol/L [27]. However, as CKD progresses, decreased kidney function prevents the kidneys from excreting enough phosphorus to maintain homeostatic balance and serum levels at adequate amounts [18]. Therefore, patients with CKD often suffer from hyperphosphatemia, which leads to serious pathogenic consequences, such as renal osteodystrophy, cardiovascular and soft tissue calcification, increased PTH and fibroblast growth factor 23 (FGF23) levels, and heart disease [17]. Furthermore, hyperphosphatemia is a strong predictor of mortality in advanced stages of CKD [27,28].

The recommended daily intake of phosphorus for the general population is 23 mmol/day (700 mg/day), with a maximum phosphorus intake level of 130 mmol/day; however, the average intake in Western countries is usually much greater. The current consensus for serum phosphorus levels is to maintain them between 3.4 and 4.5 mg/dl [29]. The most common dietary sources of phosphorus are organic phosphorus from plant foods, organic phosphorus from animal proteins, and phosphorus from additives and processed foods [22]. However, the amount of total phosphorus in food does not have a direct or proportional effect on serum levels. The bioavailability of dietary phosphorus depends on various factors, such as the form of phosphorus in food, food processing, the presence of phosphate additives, interactions between nutrients, and biological factors such as vitamin D status and bone turnover [30].

Based on this review, industrialized products and phosphate additives have a high phosphorus bioavailability of 90–100%, animal-based foods show an intermediate bioavailability of 40–80%, and plant-based foods show a bioavailability of 40–50%, although some studies report values as low as 20–40% bioavailability [7,8] (Table 1).

The most bioavailable form of phosphorus (90–100%) is in inorganic phosphate salts, which are commonly added to processed foods and beverages [31]. This is one of the reasons why high consumption of UPFs may be associated with an increased risk of chronic diseases, including decreased kidney function [32]. The phosphorus bioavailability of the latter is much lower than that of the previous sources because humans lack phytase, and, therefore, cannot breakdown and then fully absorb phytates from plants [17,31]. This low bioavailability calls into question the general recommendations for patients with CKD to avoid whole grains [32].

Additionally, studies suggest a beneficial effect of plant-based diets in slowing the progression of CKD and reducing CVD in patients with CKD [33,34], although it is unclear which nutrients have the most significant effect. A study comparing vegetarian and meat-based diets in patients with CKD revealed that a vegetarian diet resulted in lower serum phosphate and FGF23 levels after one week. This improvement may have been due to the lower bioavailability of phosphorus from plant sources, as only a fraction of the phosphate bound to phytate in plants is absorbed, even though plant-based diets do not completely restrict phosphorus absorption [14,20,30]. However, not all plant-based foods are safe for patients with CKD, as evidence suggests that the bioavailability of phosphorus from foods high in phytates is at least 50% in patients with CKD, indicating that plant-based diets do not completely restrict phosphorus absorption [28,30].

Other dietary factors that do not depend exclusively on the food itself also influence the bioavailability of phosphorus for the individual. Thus, a vegetarian diet rich in dietary fiber can reduce intestinal phosphate absorption, and some culinary preparations, such as boiling, can help reduce the bioavailable phosphate provided by the diet [27]. Soaking, sprouting, and hulling can reduce phytate levels, and boiling and draining cooking water can greatly reduce phosphorus and potassium if the liquid is discarded [28,31]. Additionally, a strategy for phosphorus control is to select foods with a favorable phosphorus-to-protein ratio to maximize protein intake and minimize the phosphorus load [35]. Recent studies have revealed that bread is an important source of phosphorus in the diet, although this food typically contains relatively low amounts of phosphorus [36].

A study comparing plant-based and animal-based diets revealed that patients with stage 3–4 CKD who followed a vegetarian diet for 7 days presented significant reductions in serum phosphorus and FGF23 levels, whereas a meat-based diet increased the levels of both markers [31].

## 4. The Role of Potassium in the Diet of Chronic Kidney Disease Patients

Potassium is the most abundant intracellular ion in the human body, with a concentration of approximately 98%, and is essential for many biological functions, such as acid–base homeostasis, cardiac function, neuronal transmission, muscle contraction, and glucose metabolism [37]. Potassium intake in patients with CKD should be adjusted according to the stage of the disease and individual response, with the following specific recommendations: at least 104 mmol/day in stages G1–G2, between 54 and 103 mmol/day in stages G3–G4, between 78 and 104 mmol/day for patients on peritoneal dialysis, and between 70 and 78 mmol/day for patients on hemodialysis.

Patients with CKD are usually at high risk of developing elevated serum potassium levels, known as hyperkalemia, because the kidney’s ability to excrete potassium decreases [37]. As a result of this increase, hyperkalemia can cause cardiac arrhythmias and is a major concern for patients with CKD [38]. For this reason, 87.1% of patients with CKD take angiotensin-converting enzyme inhibitors or angiotensin receptor blockers to control hypertension, which requires limiting potassium intake to keep serum potassium within a safe range [38].

In patients with CKD, increased potassium intake is estimated to cause approximately 11,000 additional deaths from CVD, with 42% of these deaths attributed to hyperkalemia in people with renal failure [39]. Therefore, patients with CKD are advised to limit their dietary potassium intake to maintain serum levels within 3.5–5.5 mEq/L [18]. Nutrition guidelines for CKD recommend vegetables and fruits that are low in potassium and high in fiber, as well as boiling vegetables and culinary treatment, to decrease their potassium content [40]. In example, a previous work demonstrated that soaking foods in a ratio of five parts water to one part foods for 5–10 min effectively reduced the potassium by 40–49% in beef, green leafy vegetables, and grains and between 10 and 30% in other foods [41].

To regulate potassium intake, a diet rich in fruits and vegetables can be beneficial in the early stages of CKD, both for its potassium content and for reducing the acid load [27]. On the other hand, many products marketed as “healthy” because they contain low levels of sodium often contain potassium chloride as a substitute for sodium salt, which further complicates potassium control [14].

## 5. The Role of Sodium in the Diet of Chronic Kidney Disease Patients

The effect of sodium on the progression of CKD is related to its central role in controlling water–salt homeostasis [17]. Sodium intake has a positive effect on improving nutritional status and a negative effect on nutritional status by increasing fluid volume, which causes hypertension and increases proteinuria. In CKD patients who are not on dialysis, clinicians must consider the balance between the positive and negative effects of sodium restriction. In dialysis patients, the nutritional benefits of sodium intake often outweigh the negative effects [42]. In addition, sodium excretion is related to blood pressure. Increased sodium excretion in the kidneys requires elevated glomerular pressure, which also causes hypertension [43]. In CKD, hypertension is common and constitutes an important cardiovascular risk factor. Its prevalence reaches 80–100% in advanced stages of CKD. Sodium intake, which is almost completely absorbed in the gut, should be limited to less than 87 mmol/day to reduce CVD and blood pressure risk.

There is strong evidence of the effectiveness of low-sodium diets for the treatment of CKD. Excessive dietary sodium is a major risk factor for hypertension and CVD, which are the leading causes of CKD worldwide [39]. To control hypertension, reducing sodium intake to less than 2 g/day is one of the most cost-effective strategies for improving overall health outcomes. Sodium restriction has also been shown to reduce extracellular fluid volume and improve the efficacy of renin–angiotensin–aldosterone system (RAAS) inhibition. It can reduce the need for antihypertensive medications and decrease proteinuria, slowing the progression of CKD [44]. For this reason, the 2020–2025 Dietary Guidelines for Americans recommend a daily sodium intake of 2.3 g (5.8 g of salt), whereas the American Heart Association recommends 1.5 g (65 mmol of sodium or 3.8 g of salt) for high-risk individuals, including those with CKD [42]. For patients with CKD, the 2024 KDIGO clinical practice guidelines for blood pressure management recommend a maximum of 2 g of sodium or 5 g of NaCl per day in patients with CKD and hypertension. However, sodium restriction is not recommended in patients with CKD and salt-losing nephropathy [43].

Effective habits for reducing sodium intake can be acquired by identifying and eliminating foods with high sodium content from the diet, such as processed foods, canned vegetables, pickled and fermented foods, soups, potato chips, and salted nuts and seeds [17]. Foods that have been reformulated to include lower sodium contents often use potassium-enriched salt substitutes in their manufacture, which can be harmful in patients with advanced CKD, causing hypoaldosteronism or other conditions that alter potassium excretion [44]. Notably, sodium restriction in patients with CKD can also cause nutritional imbalances and hyponatremia [43], and is often challenging because of factors such as psychological distress, taste alteration, limited access to fresh foods, and comorbidities [44], which often hinder long-term compliance with prescribed measures [42,44].

In terms of dietary recommendations, the DASH diet and the Mediterranean diet are the most frequently recommended dietary patterns for these patients, with the DASH diet generally showing better results in terms of blood pressure reduction [44].

## 6. The Role of Proteins in the Diet of Chronic Kidney Disease Patients

With respect to protein intake, as was stated previously, the KDIGO 2024 Clinical Practice Guidelines for Nutrition in CKD patients recommend 0.8 g/kg/body weight/day of dietary protein for healthy adults, whereas for patients with CKD, this value depends on the stage of the disease, which is determined by the decline in the glomerular filtration rate [13,18]. Attention should be given to the type of protein source prescribed to these patients. Animal protein sources are linked to high plasma levels of uremic toxins produced by the GM, such as indoxyl sulfate (IS), p-cresyl sulfate (pCS), and trimethylamine n-oxide (TMAO), which are associated with a high risk of CVD. Some deep-sea fish (or seafood), such as lobsters, shreds, snow crabs, and cod, have high muscular TMAO contents that, when consumed, increase systemic TMAO plasma levels [19]. However, plant-based proteins do not have negative effects on CKD patients, who often purchase animal-origin sources of proteins [14].

A low-protein diet (LPD) has been shown to improve hyperfiltration, reduce nitrogenous waste, and alleviate renal workload by decreasing glomerular pressure [17]. One study reported that proteinuria decreased by 20% to 50% in CKD patients who followed an LPD [20]. However, although LPDs are beneficial for CKD patients, healthcare professionals have not reached a complete consensus on their benefits, as this type of diet also has some harmful effects on CKD patients. These negative effects are due to possible malnutrition and the waste of protein and energy due to inadequate energy intake [20]. Therefore, considering the potential benefits and harms of LPDs, an intake of 25–35 kcal/kg/body weight/day is recommended to maintain energy and nitrogen balance and avoid the risk of malnutrition [17].

## 7. Influence of Vegetable and Dietary Fiber Intake on Inflammation Biomarkers and Chronic Kidney Disease

There is ample scientific evidence demonstrating the impact of diet on inflammatory pathways. In relation to this, it is known that certain food components, such as saturated fats, refined sugars, processed foods, and UPFs, have pro-inflammatory properties that can contribute to a low-grade systemic inflammatory state [2]. Conversely, diets high in vegetables, whole grains, and healthy fats have been linked to anti-inflammatory effects on the human body [2,31]. Additionally, a plant-based diet has been shown to reduce metabolic acidosis and blood pressure, decrease markers of kidney damage, reduce body weight, and improve cardiovascular outcomes [45].

Historically, the consumption of fiber-rich fruits and vegetables has been restricted in patients with CKD to combat hyperkalemia [33,46]. Among the reasons often given for this restriction are concerns about their high potassium and phosphorus contents. However, essential aspects related to their bioavailability have not been adequately assessed [9]. Recent findings suggest that the intake of fibers, especially insoluble fibers, may promote potassium excretion through the intestine, which could help control the risk of hyperkalemia in patients on maintenance hemodialysis [47]. This effect is primarily mediated by the ability of insoluble fibers to increase fecal bulk and enhance intestinal transit, which facilitates the binding of potassium ions within the gut lumen. Insoluble fibers, such as cellulose and hemicellulose, are poorly fermented by colonic bacteria and are not digested by microorganisms in the large intestine, usually found in the roots, stems, dry leaves, peels, and fruit of plants. They retain water, increasing stool volume, which stimulates colonic potassium secretion via active transport mechanisms in the distal colon, while also promoting the trapping of potassium in the stool, thereby increasing fecal excretion [47]. This increase in fecal bulk also improves constipation, which is common in patients on maintenance hemodialysis, and reduced intestinal excretion of potassium due to constipation may be an important cause of pre-dialysis hyperkalemia. Additionally, insoluble fibers may indirectly modulate potassium absorption by altering the gut microbiota composition, favoring bacterial populations that metabolize compounds affecting electrolyte balance. Collectively, these processes reduce systemic potassium levels, providing a protective effect against hyperkalemia in patients with CKD [47].

The bioavailability of phosphorus varies depending on its origin and is lower in vegetables than in other foods because phosphorus is present in the form of phytate. Therefore, the impact of high-fiber plant foods, such as whole grains, on phosphorus exposure is minimal for most patients [30]. Even so, most patients with CKD currently consume less than the recommended amount of dietary fiber, which has an impact on their nutritional health [1]. The consumption of plant proteins produces alkalizing and anti-inflammatory effects that preserve kidney function, whereas the metabolism of animal proteins generates an acidic environment and reactive species that damage the glomeruli [31]. It has also been reported that a diet high in plant-based proteins may have a protective effect on preserving glomerular function (GFR) and reducing albuminuria. An experimental study in an animal model revealed that diets rich in plant proteins can reduce the expression of the renin–angiotensin system and slow the progression of CKD in animal models [14]. In addition, a low protein-to-fiber ratio, as is typical of diets that include many plant foods, causes patients to have lower serum levels of phosphorus and potassium [2].

In addition to the effects of high-fiber plant foods related to their concomitant phosphorus and potassium contents, dietary fiber intake also has other effects on human health that are worthy of consideration. Among these, CVD is the leading cause of death in the CKD population. A high ratio of dietary protein to fiber is associated with an increased risk of developing CVD [48]. Notably, CKD can also be considered an inflammatory state, and high fiber intake has been associated with reduced inflammation in the general population [49]. In fact, a clinical study based on data from 14,543 participants in the National Health and Nutrition Examination Survey III revealed that for every 10 g/day increase in total fiber intake, an 11% decrease in C-reactive protein (CRP) levels was observed in individuals without CKD and a 38% decrease in patients with CKD. These findings suggest that increasing fiber intake may help reduce inflammation and cardiovascular risk in patients with CKD.

In a large cohort of adults with CKD, higher dietary fiber intake was associated with lower mortality in people with CKD [33], regardless of inflammatory biomarker levels, history of CVD, and degree of renal dysfunction. Furthermore, a systematic review and meta-analysis of controlled trials on diet in individuals with CKD revealed a reduction in serum urea and creatinine with dietary fiber supplementation. In total, 12 of the 13 trials included fermentable fiber, further supporting the role of colonic bacteria in improving renal outcomes [50]. Similarly, Cui et al. [1] investigated the impact of soluble dietary fiber on biochemical parameters in non-dialyzed CKD patients. Intervention with soluble dietary fiber significantly reduced total serum cholesterol levels and plasma interleukin-8 (IL-8) levels, which are key inflammatory markers. In addition, fiber intake increased by 18.1 g/day after supplementation with soluble dietary fiber, contributing to a positive effect on the inflammatory process. Inflammatory markers such as CRP, tumor necrosis factor-α, fibrinogen, procalcitonin, and white blood cell count showed significantly lower levels in a group of people with a low dietary inflammatory index, which included high fiber intake [2]. An elegant recent review showed that dietary fiber intake reduced interleukin-6 and tumor necrosis factor-α, although it did not reduce high-sensitivity CRP [51]. The reduction in inflammatory markers because of increases in dietary fiber intake seems to be stronger in patients with CKD than in healthy individuals [51].

Inflammation in CKD is also related to chronic metabolic acidosis, which is associated with elevated CRP levels. This condition is exacerbated by a low intake of fruits and vegetables, which are essential sources of dietary alkali [49].

A high intake of fruits and vegetables, in addition to providing high dietary fiber content, also provides a high intake of alkalis, recommended to reduce metabolic acidosis [3]. Metabolic acidosis, which worsens with the progression of CKD, aggravates the course of CKD by promoting insulin resistance and increasing oxidative stress and inflammation, leading to protein loss and malnutrition [3]. Metabolic acidosis frequently results in worsening renal function, promotes decreased estimated glomerular filtration (eGFR), and contributes to extrarenal complications [3]. The consumption of plant-based foods helps neutralize acid via citrate. Thus, the consumption of plant-based proteins may have alkalizing and anti-inflammatory properties, which help preserve kidney function [31]. In patients with an eGFR < 60 mL/min/1.73 m^2^, a diet with a higher proportion of plant-based protein has been associated with lower all-cause mortality [38].

A cohort study conducted by Mirmiran et al. [4] revealed that people who consumed more than 26 g/day of fiber had a 50% lower risk of developing CKD than those who consumed ≤ 17.7 g/day of dietary fiber. In addition, the study reported an 11% reduction in the incidence of CKD for every 5 g/day increase in total fiber intake. The study also revealed that women with no history of kidney stones who consumed the highest amounts of dietary fiber had a 22% lower risk of developing kidney stones than those with the lowest intake [4].

## 8. Gut Microbiota and the Gut–Kidney Axis in Chronic Kidney Disease

Plant-based diets are rich in dietary fiber, which plays a key role in modulating the GM and reducing the production of uremic toxins. Bidirectional communication occurs through the gut–kidney axis and involves the exchange of metabolites and signaling molecules [51]. In contrast, animal-based diets promote the formation of proteolytic fermentation products (phenols, indoles, amines, and ammonia), which contribute to intestinal dysbiosis and the accumulation of toxins [9]. Bacterial species from the GM convert choline and L-carnitine derived from meat, fish, dairy products, and eggs into trimethylamine, which is oxidized in the liver to form TMAO. This proinflammatory compound is associated with increased cardiometabolic risk in adults with CKD [14].

The consumption of plant-based proteins favors the production of short-chain fatty acids (SCFAs), which help maintain intestinal integrity and reduce inflammation. Scientific reviews highlight the role of plant-based diets in mitigating complications of CKD, such as hyperphosphatemia, hypertension, metabolic acidosis, uremic toxemia, hyperlipidemia, diabetes, and kidney stones [9]. Among SCFAs, butyrate is known as the main energy source of enterocytes [12]. Butyrate can be derived directly from the diet (minor source) or endogenously from intestinal bacteria through the fermentation of indigestible dietary fiber (major source) [51].

Dietary fiber plays a role in reducing the production of uremic toxins, preserving kidney function, and slowing the progression of CKD through metabolic, immunological, and inflammatory regulation [52]. In addition, fiber intake has been linked to reduced CRP levels in an experimental model of CKD in mice fed a high-fat diet with fiber supplements [9].

Tomova et al. [53] demonstrated that increased fiber intake is associated with anti-inflammatory effects and improved GM composition, which could contribute to the differences observed in inflammatory markers and renal function between groups [53]. Dysregulation of the GM has been shown to worsen kidney disease [51]. Compounds such as claudin-1 and occludin are key elements in the intestinal epithelial barrier and decrease in CKD, contributing to disruption of the epithelial junction [54].

Other works have reported important effects of dietary fiber intake in CKD patients, such as preventing CKD events [21], maintaining improvements in the eGFR and urinary albumin-to-creatine ratio (ACR) [24], reducing proinflammatory cytokines [55], lowering the risk of CVD [56], lowering the acid load [57], and lowering the risk of new-onset hypertension [58]. Table 2 shows the results of the intake of different dietary fibers and foods with high contents of dietary fiber as whole grains in CKD patients.

**Table 2:** Effects of dietary fiber intake in CKD patients.

Type of Work	Subjects	Intervention	Main Effect on Kidney	Reference
Randomized controlled study	40 CKD patients in stage 3–5 CKD, between 18 and 80 years	Supplementation with 15 g/day soluble dietary fiber for 30 days in non-dialyzed patients with advanced CKD	Supplementation with soluble dietary fiber for 30 days in non-dialyzed patients with advanced stage CKD reduced total cholesterol and interleukin-8 levels (a marker of inflammation). In addition, it increased the production of propionate, a beneficial metabolite, although it did not change microbial diversity. Fiber intake increased by 18 g daily, showing benefits in biochemical, inflammatory, and metabolic parameters.	[1]
Cross-sectional trial	14,543 subjects > 20 years old	National Health and Nutrition Examination Survey III with eGFR < 150 mL/min/1.73 m^2^	High fiber intake in people with CKD not only decreases inflammation, but is also associated with lower mortality. CKD is an inflammatory state, characterized by elevated levels of C-reactive protein (CRP).	[50]
Prospective Cohort Study	1630 subjects (for 6–1 years) free of CKD	Dietary fiber intake from various sources was assessed by frequency questionnaire in 1630 CKD-free participants	Fiber intake, mainly from legumes and vegetables, was inversely associated with the risk of CKD incidence.	[4]
Prospective Cohort Study	470,778 (aged 40–69 years) participants	11-year follow-up, there were 13,555 participants who developed CKD events and 457,223 participants who stayed free of CKD	High consumption of certain foods, such as whole-grain bread, oat cereal, muesli, fruits, and raw and cooked vegetables, was found to be associated with a lower risk of CKD events. In contrast, a high intake of white bread, processed cereals, processed meats, added salt, pork, chicken, beef, and lamb was associated with an increased risk of CKD.	[21]
Prospective Cohort Study	6044 subjects aged ≥18 years old	Dietary factors and their relationship with the risk of developing CKD were analyzed over a follow-up of 7.7 ± 2.7 years	Diets with a higher glycemic index were associated with a 30% higher risk of developing CKD, whereas total diets with a healthier carbohydrate score had a 15% lower risk of CKD. Participants who consumed more whole grains compared to refined grains had a 19% lower risk of developing CKD.	[45]
Prospective Cohort Study	3787 participants between 26 and 65 years of age	Nutritional follow-up over a 15-year period, with evaluations performed at least three times, each five years apart	Consumption of fruit and vegetables was not associated with changes in estimated glomerular filtration rate (eGFR) and urinary albumin to creatine ratio (ACR).	[24]
Randomized controlled study	120 middle-aged and older participants	Whole grains (WGs) or refined grains were provided as standardized staple foods (150 g rice + 150 g flour per day) to 120 middle-aged and older adults for 6 weeks, with daily intake recorded using dietary diaries and electronic scales	WG intake significantly reduced proinflammatory cytokines IL-22 and IL-23, associated with changes in short-chain fatty acids and CD4+ T-cell subsets. The anti-inflammatory effects of whole grain were confirmed to be related to T-cell modulation.	[55]
Cross-sectional study	3110 subjects	Frequency of WG consumption assessed via validated food frequency questions based on national dietary guidelines	Regular intake of WG is associated with a lower risk of CVD, especially hypertension. Due to its benefits, it is recommended to promote access to and consumption of WG in food policies to prevent these diseases.	[56]
Cross-sectional study	16,325 participants from NHANES, National Health and Nutrition Examination Surveys	WG and refined grain consumption were assessed using dietary questionnaires for 3 months	WG intake was associated with an increase in estimated glomerular filtration rate and a decrease in urinary albumin–creatinine ratio. Consumption of refined grains had adverse effects. In addition, participants with higher WG intake showed lower uric acid levels, which is related to a lower risk of cardiovascular disease and CKD.	[59]
Comparative descriptive study	Not applied	Evaluation and comparison of the nutritional composition of different diets in a direct way, using specific meal plans adapted to patients with CKD	Vegetarian renal diets may be especially beneficial because they provide high amounts of dietary fiber and K1 vitamins, thus, they benefit renal function by lower acid load.	[57]
Retrospective cohort	10,973 participants without hypertension	WG intake was assessed by 3 consecutive 24 h dietary recalls combined with a household food inventory	High consumption of WG was associated with a lower risk of new-onset hypertension.	[58]
Cross-sectional study	3791 chronic renal insufficiency	Self-reported dietary fiber intake was compared with hazard ratios or occurrence of all-mortality, cardiovascular, and kidney events	No relevant association was found between dietary fiber intake and all-cause mortality, adverse cardiovascular, or kidney events.	[33]
Retrospective comparative study	145 chronic kidney disease patients	Dietary inflammatory index compared with clinical data	Patients with low dietary inflammation index (high dietary fiber and low protein intake) showed significantly lower serum creatinine, phosphorus, and potassium, as well as higher hemoglobin levels compared to high dietary inflammation index.	[2]
Cross-sectional study	5094 adults	Validated food frequency questionnaire and transversal comparison with health status	Higher WG intake was associated with better diet quality in both sexes. In men, higher WG consumption was associated with lower body mass index, smaller waist circumference, and lower total cholesterol. These effects were not observed in women.	[60]

## 9. Functional Compounds of Vegetable Origin with Activity in CKD Patients

In addition to their dietary fiber content, vegetable foods are well known for containing several bioactive compounds, some of which play important roles in reducing reactive oxygen species (ROS) production, metabolic stress, and inflammation [61]. Among the bioactive compounds from vegetable foods, many potent flavonoids can be found in fruits and vegetables [23]. The intake of vegetables with high contents of these bioactive compounds can reduce oxidative stress and inflammation, modulating biochemical pathways such as the expression of the nod-like receptor pyrin domain containing 3 (NLRP3) inflammasome, a transcription factor involved in the inflammatory response [23]. Additionally, many bioactive compounds found in vegetables and fruits activate cytoprotective transcription factor 2-related erythroid 2 (Nrf2), a protein that regulates the expression of antioxidant proteins that protect against oxidative damage caused by injury and inflammation [23]. All results shown regarding the activity of bioactive components should be taken with due caution, as they correspond to preclinical trials or have been obtained in experimental animals.

In experimental models of kidney injury, fisetin, a flavonol found in several vegetables, inhibits the renal production of proinflammatory cytokines and proteins related to kidney inflammation [62]. The specific beneficial effects of flavonoids on kidney function were also found for other flavonoid compounds, such as quercentin, apigenin, and phloterin. The protective effects of quercetin, a flavonol that can be found in several vegetable foods [63], on kidney injury, including improvements in renal function; protection against vascular calcification; and reductions in oxidative stress, serum FGF23 levels, and renal inflammation, have been demonstrated in animal models [64]. Apigenin also has antioxidant properties, and in a mouse model of induced kidney injury, apigenin administration mitigated kidney damage, in addition to reducing the levels of serum creatinine, blood urea nitrogen, glutathione peroxidase, and superoxide dismutase [65]. Another flavonoid is phloretin, which has antioxidant effects that include scavenging free radicals and reducing lipid peroxidation, thus inhibiting the expression and secretion of several proinflammatory agents [23]. In an animal model of hyperuricemia-induced kidney injury, phloretin, in addition to improving kidney function, reduced the levels of NLRP3 and interleukin-1β (IL-1β), mitochondrial ROS, and morphological lesions in the kidney [66].

A less-known bioactive compound that can be useful for CKD patients is allicin. Allicin is a compound present in vegetable species of the genus Allium, such as white garlic, and is a volatile sulfur-containing compound [67]. Allicin has antioxidant effects by producing S-allyl-mercapto-glutathione from reduced glutathione [68]. Studies in rats have shown that allicin contributes to regulating hypertension and can also improve renal function, reduce oxidative stress, and upregulate the expression of the Nrf2/Kelch-like ECH-associated protein 1 (keap1) pathway [69].

Sulforaphane is an oily sulfur-containing isothiocyanate derived from glucoraphanin that occurs naturally in cruciferous vegetables from the Brassicaceae family, such as broccoli [23]. Its effects are derived from different pathways, although its best-studied mechanism related to kidney health is its stimulatory effect on the Keap1–Nrf2 pathway. However, there are no studies on the effects of this bioactive compound in CKD in vitro, and animal studies have shown that it has beneficial effects on kidney health by reducing DNA damage, increasing the synthesis of phase II antioxidants via phosphatidylinositol 3-kinase (PI3K)/Akt-mediated transcription factor Nrf2 activation, and reducing oxidative stress [70].

Proanthocyanidins (PACs) are polyphenolic compounds that are oligo- and polymeric end products of the flavonoid biosynthesis pathway in plants [23]. They are commonly present in plants and fruits, nuts, seeds, pine bark, and berries, which contain the highest levels of PACs. A recently randomized, double-blind, placebo-controlled pilot study demonstrated that supplementation with 500 mg of dry cranberry extract twice daily for two months did not reduce the plasma levels of lipopolysaccharides (LPSs) or uremic toxins produced by the GM in nondialysis CKD patients [71].

Although no in vitro or in vivo trials have specifically investigated its effects on patients with kidney damage, other compounds found in vegetables with potent antioxidant activities are expected to have beneficial effects on CKD patients. Among them, epigallocatechin gallate (EGCG), caffeine, chlorogenic acid and kahweol from coffee, polyphenols from cocoa, epicatechin from chocolate, and resveratrol from grapes and red wine can be found in green tea [23].

Ginger (*Zingiber officinale*) contains several bioactive compounds, including shogaols, gingerols, gingerdione, capsaicin, pinene, and zingiberol, with gingerol being the most abundant [23]. Ginger appears to be beneficial in improving oxidative stress and inflammation parameters through the suppression and modulation of several pathways, such as NF-κB translocation, the NLRP3 inflammasome, and Nrf2 [72]. Streptozotocin-induced diabetic rats treated with 6-gingerol presented increased levels of fasting glucose, hyperlipidemia, malondialdehyde, inflammatory markers, NF-κB protein expression, and antioxidant enzymes in the kidneys, and there was still improvement in kidney damage [73]. Other compounds, such as zingerone [74] and 6-shogaol [75], have shown protective antioxidant and anti-inflammatory effects in animal models of kidney injury.

In addition to their metabolic effects due to their antioxidant or anti-inflammatory properties, bioactive compounds from vegetable foods can also modulate the composition and functionality of the GM, but micronutrients can also significantly affect the composition and functionality of the GM [76]. Thus, some of the cited bioactive compounds, such as polyphenols, can increase the abundance of gut *Bifidobacterium* and the synthesis of metabolites that mitigate inflammation [23]. Additionally, *Lactobacillus* species use polyphenols as substrates to produce alkyl catechols, which are well-established Nrf2 agonists [72].

## 10. Cereal-Based Food Intake in CKD Patients

Whole grains (WGs), such as brown or wild rice, quinoa, oats, couscous, and WG pasta and bread, are healthy sources of carbohydrates and essential nutrients. In contrast, the food industry commonly uses refined grains, which lack fiber and essential nutrients and contain more preservatives, in their formulations [14]. A recent intervention study demonstrated that the inclusion of parsley seed bread in a balanced, low-calorie diet improved anthropometrics, blood pressure, lipid profile, osteopontin, IL-1β, IL-10, and kidney function (creatinine, eGFR, and creatinine clearance); these benefits decreased after switching to Baladi bread [77].

The regular consumption of WG has been linked to decreased blood pressure, reduced cardiovascular risk factors, and improved metabolic health. These benefits are independent of other sources of dietary fiber, suggesting the presence of additional cardioprotective compounds [56]. In addition, WG consumption is inversely associated with body mass index (BMI), waist circumference, and total cholesterol in men [60]. A recent study revealed that higher WG consumption was associated with lower serum uric acid levels, whereas refined grain consumption was positively associated with uric acid concentrations, which are associated with increased cardiovascular risk and CKD progression [59].

In a recent work, Li et al. [55] compared the effects of WG consumption and refined grain consumption on immune-mediated inflammation. Compared with refined grain consumption, WG consumption significantly reduced the circulating levels of the proinflammatory cytokines IL-22 and IL-23. These reductions were associated with optimized SCFA profiles and changes in CD4+ T-cell subsets, suggesting that the anti-inflammatory effects of WG occur through both modulation of the GM and regulation of the immune system.

In another work, Winkelman et al. [30] investigated the appropriateness of the traditional recommendation to avoid WG in patients with CKD. This study highlights concerns about the increased consumption of UPF derived from plants, particularly industrial meat and dairy substitutes, which are associated with poor cardiovascular and renal outcomes, among vegetarians and vegans. Increased intake of UPF has also been linked to increased morbidity and mortality from obesity, type 2 diabetes, hypertension, cerebrovascular disease, and coronary heart disease, including decreased kidney function, in populations without CKD [32].

One of the most widely consumed cereal-based foods in the Western world is bread, which can be made with refined flours or whole grain flour. In addition to the type of flour used to make it and its resulting fiber content, bread is also very important for patients with CKD because it is one of the foods with the highest salt content, meaning that it contributes significantly to sodium intake [78]. One of the most used methods to reduce sodium intake in the Western population is to reformulate the type of salt used in bread making. In fact, bread is the food most subject to salt reduction worldwide, followed by other bakery products, processed meats, sauces, and prepared foods [79]. In this regard, it is common to replace sodium chloride (NaCl) with potassium chloride (KCl) [39]. In addition to the drawback of high sodium content, bread can also pose a risk to CKD patients because some phosphorus additives present in bread, despite being less than 2% by weight, still represent a significant portion [13]. However, it should be considered that the phytase-fortified flours used for some breads, aimed at increasing the bioavailability of minerals of Fe or Zn [80], may also increase the bioavailability of phosphorus in a secondary manner, which makes these flours less suitable for patients with CKD.

A cross-sectional study investigated the impact of replacing NaCl in bread with KCl at proportions of 20%, 30%, and 40%. This substitution caused one-third of patients with CKD to exceed safe dietary potassium limits, highlighting the need for mandatory food labeling to prevent excessive potassium intake [39]. Another study, which compared phosphorus and phosphate regulatory factors in people with different patterns of bread, rice, and rice noodle consumption, revealed that the group with high noodle consumption had significantly increased serum phosphorus levels compared with the groups with high bread and rice consumption. This was attributed to the fact that instant noodles contain additional food additives containing phosphorus, which are used as thickeners, humectants, and colorants [36]. In Table 3, the main effects of different bread types consumption on kidney function can be found.

**Table 3:** Effects of different bread types on kidney function and dietary exposure.

Type of Work	Subjects	Intervention	Main Effects On Kidney	Reference
Clinical Trial	85 obese women (BMI: 35.68 ± 0.47 kg/m^2^)	8-week nutritional intervention with two phases: Phase 1 (4 weeks): 100 g/day of ground parsley seed bread along with a healthy balanced low-calorie diet. Phase 2 (4 weeks): parsley seed bread replaced with Baladi bread providing equivalent caloric intake. Anthropometrics, blood pressure, dietary recalls, and biochemical parameters (osteopontin, IL-1β, IL-10, kidney function, and lipid profile) were measured before and after each phase. Low-calorie diet	Parsley seed bread with a healthy diet improved anthropometrics, blood pressure, lipid profile, osteopontin, IL-1β, IL-10, and kidney function (creatinine, eGFR, creatinine clearance); benefits decreased after switching to Baladi bread	[77]
Cross-sectional study	109 healthy people	Subjects were categorized into groups based on consumption frequency of rice, bread, and noodles	Serum levels of phosphorus and phosphate-regulating factors did not differ according to bread consumption	[36]
Cross-sectional study	12,152 subjects, from which 154 were CKD patients	Replacement of sodium in bread with 20%, 30%, or 40% KCl; dietary intake assessed via two 24 h recalls in 12,152 subjects (including 154 CKD patients)	Substitution of sodium for KCl in bread products resulted in one-third of people with chronic kidney disease (CKD) exceeding safe limits for potassium intake, with values of 31.8%, 32.6%, and 33% for 20%, 30%, and 40% substitutions, respectively	[39]
95 American bread types	Not applied	Analysis of bread composition	Most of the breads analyzed did not mention the use of phosphates as additives, or the information was not available. Of the 95 breads reviewed, 7 (7.4%) contained phosphorus additives, although in amounts less than 2% of the total weight. In addition, 11 breads (11.6%) had these additives in small amounts, and 4 breads (4.2%) included them as part of the leavening nutrients or dough conditioners	[13]

## 11. Resistant Starch Intake in CKD Patients

Starch is a polymeric carbohydrate produced by most plants for the purpose of storing energy [81]. Resistant starch (RS) is composed of glucose monomers linked by α-glycosidic bonds in the form of amylose and amylopectin polymers [82]. These bonds are resistant to hydrolysis in the human small intestine because they are physically or chemically resistant to digestive α-amylases [83]. In recent years, RS has gained popularity in the world of nutrition and has been promoted as an ally for weight loss and digestive health. RS reaches the colon, where it is metabolized by certain types of bacteria in the GM, generating beneficial metabolites such as SCFAs. This type of starch is also associated with a greater feeling of satiety. By reducing the production of leptin, a hormone that regulates appetite, the body receives signals of fullness more quickly, which can lead to the consumption of fewer calories throughout the day [84].

**Figure 3:**
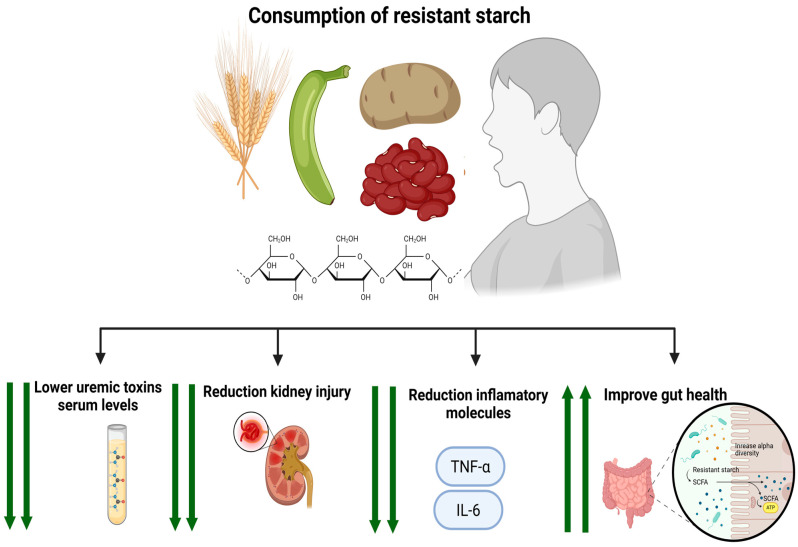
Effect on resistant starch consumption in patients with CKD.

In addition, RS intake also provides benefits for patients with CKD (Figure 3). In this context, Tayebi Khosroshahi et al. [85], in a randomized controlled trial with hemodialysis patients, demonstrated that introducing RS into the diet could effectively reduce systemic inflammation and renal burden. The benefits of RS supplementation in reducing systemic inflammation have been demonstrated in patients with CKD and in animal models. For example, Tayebi Khosroshahi et al. [85] and Laffin et al. [86] reported significant decreases in the levels of tumor necrosis factor-alpha and IL-6, which are key inflammatory markers, in patients undergoing hemodialysis after RS supplementation (Table 4). Similarly, Vaziri et al. [87] reported a reduction in the activation of the NFkB pathway, which is associated with oxidative stress and inflammation, in rats with CKD that received RS supplements. These reductions in inflammation indicate the potential of RS as a nonpharmacological approach to treat proinflammatory and immune responses in the kidney and improve the progression of CKD. Moreover, RS supplementation in individuals with CKD attenuated the concentrations of uremic retention solutes, including IS and PCs [88]. Many uremic toxins, such as ISs and PCs, are produced entirely by the GM [54,89] through the proteolytic digestion of aromatic amino acids (tyrosine and tryptophan, respectively) [90]. As a result of RS supplementation and the subsequent shift in microbial fermentation to the distal colon, protein fermentation is reduced, partly due to a general preference for carbohydrates over amino acids as an energy source for gut microbes [91]. The decrease in proteolytic fermentation subsequently reduces the levels of supposedly harmful and proinflammatory metabolites, such as PCs [91]. Zhang et al. [82] reported that RS supplementation can improve symptoms associated with the presence of uremic toxins and renal function in patients with CKD. In these patients, blood IS and blood urea nitrogen (BUN) levels are decreased [82], whereas no significant differences in other markers are detected. BUN is important because as kidney function deteriorates with the progression of CKD, urea levels increase in the individual’s intestinal lumen [91]. Consequently, urea is metabolized by specific bacteria that express urease in the colon, which converts urea into ammonia products that damage the tight junctions between colon epithelial cells, thereby increasing intestinal permeability [91]. In addition, certain colon bacteria are the source of key uremic toxins that contribute to damage to the epithelium lining the intestine and are associated with increased mortality and progression of CKD [91,92,93].

In addition to causing a decrease in protein fermentation rates and its effects on the metabolism of nitrogenous components, RS also exerts other types of actions on the GM that are worth mentioning. The specific mechanisms by which RS affects the GM, such as increasing the production of beneficial SCFAs, improving the integrity of the intestinal barrier, and reducing the levels of uremic toxins, are directly relevant to improving outcomes in patients with CKD [54]. CKD is characterized by a change in the composition of the GM, with an increase in Firmicutes, Clostridiaceae, and Enterobacteriaceae bacteria and a reduction in beneficial *Bifidobacterium* and *Lactobacillus* bacteria [92]. In patients with CKD, dysbiosis is associated with inflammation, oxidative stress, and ROS production. Together, these factors cause further kidney damage by microcirculation and blood perfusion [82].

Animals with CKD showed marked depletion of colonic claudin-1 tissue and occlusion, which are key components of tight epithelial junctions, while consumption of an RS-enriched diet significantly restored claudin-1 expression. These observations generally support the role of RS in reducing inflammation and improving renal and intestinal epithelial barrier function, which is consistent with the delay in the progression of CKD [87].

A meta-analysis by Wu et al. [94] demonstrated that dietary fiber supplements could significantly reduce plasma PC levels in patients with CKD, and Khosroshahi et al. [85] reported that PC levels were significantly reduced in end-stage renal patients after treatment with RS. However, Esgalhado et al. [95] reported opposite results. However, the reductions in p-cresyl sulfate levels were inconsistent, only being found in some works [88,91]. Thus, this absence reduces the beneficial effects of RS intake for CKD patients.

**Table 4:** Effects of different types of resistant starch (RS) on CKD patients.

Type of Work	Subjects	Intervention	Main Effects on Kidney	Reference
Randomized trial	18 male Sprague–Dawley rats CKD induced	Low-fiber diet or 59% HAM-resistant starch for 3 months	Increase serum urea and creatinine levels and reduced creatinine clearance in controls. Histological analysis demonstrated that rats for RS showed reduced kidney injury compared to CKD controls	[87]
Randomized controlled trial	46 CKD with hemodialysis patients	Placebo or 20 g/day RI for 4 weeks, followed by 25 g/day for other 4 weeks	Serum creatinine and urea were significantly decreased in the RS added group with respect to placebo group	[85]
Randomized double-blind placebo controlled trial	20 CKD with hemodialysis patients	Placebo or 20 g/day RI for 4 weeks, followed by 25 g/day for other 4 weeks	Serum urea, IL-6, TNFα, and MDA were decreased among the RS patients with respect to those received placebo	[86]
Randomized double-blind placebo controlled trial	16 CKD with hemodialysis patients	16 g/day RS of placebo for 4 weeks	RS supplementation effectively reduced inflammatory molecules such as interferon-inducible protein 10 and platelet-derived growth factor	[96]
Randomized double-blind, crossover placebo controlled trial	43CKD with hemodialysis patients	21 g/day RS or placebo for 4 weeks, and vice-versa after 4 weeks washout period	Serum levels of uremic toxins were lower in RS-treated group	[97]
Randomized double-blind placebo controlled trial	68 CKD patients	15 g/day RS in week 1 and 33 g/day in weeks 2–16 of placebo for 16 weeks	Reduction in serum p-cresyl sulfate in RS added patients and significant changes in gut microbiota composition, with increases in alpha-diversity	[91]
Randomized double-blind, crossover placebo controlled trial	35 KCD patients	15 g/day or RS or placebo for 26 weeks	Reduction in uremic toxins as indoxyl sulphate and p-cresyl sulphate and CKD symptoms in RS-treated patients	[88]

## 12. Conclusions

This study highlights that, in a context where proper protein intake is not neglected and there are no comorbidities that limit their suitability, plant-based foods have positive effects on slowing the progression of CKD by improving kidney function and reducing CKD-related complications. Dietary fiber, especially from natural sources such as fruits, vegetables, and whole grains, plays a key role in reducing inflammation and improving biomarkers associated with CKD. Some bioactive compounds from vegetable foods have been reported to play protective roles in risk factors of CKD reducing ROS production, metabolic stress, and inflammation. Proper management of phosphorus and potassium levels is crucial for preventing serious complications in CKD patients, and plant-based diets have shown positive results in reducing these levels because of the lower bioavailability of these minerals in plant foods. Whole grains, which are rich in essential nutrients and fiber, are linked to better metabolic health, lower blood pressure, and a reduced risk of cardiovascular disease and CKD progression. Finally, adopting a plant-based diet that limits animal protein intake provides protective effects on kidney function by promoting a healthy acid–base balance, reducing the production of uremic toxins, and improving renal and cardiovascular outcomes, making it a promising preventive and therapeutic strategy for CKD.

Nevertheless, it is important to highlight that plant-based diets are not free of potential drawbacks. To meet protein requirements, patients may consume excessive amounts of plant proteins, which could inadvertently increase phosphorus and potassium intake, creating risks of electrolyte imbalance. Exclusive or unbalanced consumption of fiber-rich foods (vegetables, fruit, whole grains, etc.) should, therefore, not be considered a panacea. Careful dietary planning and clinical monitoring remain essential, especially in advanced CKD stages or dialysis patients.

## 13. Limitations of This Review

This work is a narrative review and, as such, is subject to several limitations. The search strategy was not systematic, which means that some relevant studies may not have been included, and the selection of studies could be subject to bias. Most of the evidence discussed comes from observational studies, cross-sectional analyses, or short-term trials, which limit the ability to draw causal conclusions.

The populations studied were very heterogeneous, ranging from general population cohorts to small groups of patients with advanced CKD, making it difficult to generalize the findings across all CKD stages. Dietary interventions and exposures varied widely, including individual nutrients (fiber, potassium, and phosphorus), specific foods (whole grains, bread, and vegetables), dietary patterns (Mediterranean and DASH), and bioactive compounds, with large differences in duration, dosage, and assessment methods.

Additionally, most studies on bioactive compounds and functional foods were conducted in animal models or in vitro, with limited clinical evidence in humans, particularly in patients with advanced CKD. This raises uncertainty regarding their effectiveness, optimal dosages, and safety in real-world settings. Interactions between diet, medications, and comorbidities were also not extensively addressed, nor were practical issues such as patient adherence, regional dietary differences, and food labeling variability.

Overall, these limitations highlight the need for more robust, long-term clinical trials and additional studies to better understand the effects of plant-based foods, dietary fiber, whole grains, and bioactive compounds on CKD progression and patient outcomes.

## Figures and Tables

**Table 1:** Phosphorus bioavailability by food source.

Food Source/Type	Phosphorus Bioavailability
Inorganic phosphate additives (processed foods, beverages)	90–100%
Animal-based foods (meat, dairy, eggs)	40–80%
Plant-based foods (legumes, grains, vegetables)	40–50% (sometimes less)

## Data Availability

No new data were created or analyzed in this study.
